# Patterning of the cell cortex and the localization of cleavage furrows in multi-nucleate cells

**DOI:** 10.1242/jcs.259648

**Published:** 2022-04-14

**Authors:** Günther Gerisch, Jana Prassler, Mary Ecke

**Affiliations:** Max Planck Institute of Biochemistry, Cell Dynamics Group, Am Klopferspitz 18, D-82152 Martinsried, Germany

**Keywords:** Contractile ring, Cortexillin, Cytokinesis, *Dictyostelium*, Mitosis, Myosin II

## Abstract

In multi-nucleate cells of *Dictyostelium*, cytokinesis is performed by unilateral cleavage furrows that ingress the large cells from their border. We use a septase (sepA)-null mutant with delayed cytokinesis to show that in anaphase a pattern is generated in the cell cortex of cortexillin and myosin II. In multi-nucleate cells, these proteins decorate the entire cell cortex except circular zones around the centrosomes. Unilateral cleavage furrows are initiated at spaces free of microtubule asters and invade the cells along trails of cortexillin and myosin II accumulation. Where these areas widen, the cleavage furrow may branch or expand. When two furrows meet, they fuse, thus separating portions of the multi-nucleate cell from each other. Unilateral furrows are distinguished from the contractile ring of a normal furrow by their expansion rather than constriction. This is particularly evident for expanding ring-shaped furrows that are formed in the centre of a large multi-nucleate cell. Our data suggest that the myosin II-enriched area in multi-nucleate cells is a contractile sheet that pulls on the unilateral furrows and, in that way, expands them.

## Abstract (precis)

**Summary:** Multi-nucleate *Dictyostelium* cells divide by unilateral cleavage furrows that progress and expand according to a pattern of cortexillin and myosin II that is determined by microtubule asters at the spindle poles.

## INTRODUCTION

In this study we explore pattern formation in the cortex of mitotic cells by placing large multi-nucleate cells between two planar surfaces. Normal mononucleate *Dictyostelium* cells divide, similar to animal cells, by forming a cleavage furrow enriched in filamentous myosin II (Fukui and Inoué, 1991; Neujahr et al., 1997; Zang and Spudich, 1998). The semi-closed mitosis of *Dictyostelium* begins with the translocation of the centrosome into the nucleus, centrosome division and the intranuclear initiation of a spindle (Gräf et al., 2021). Subsequently, the spindle elongates within the cytoplasmic space, while the segregating chromosomes remain enveloped by the nuclear membrane. The cleavage furrow begins to separate the two daughter cells after the spindle has been disassembled in the midzone. Accordingly, the position of the furrow is not determined by the spindle but by exclusion from the microtubule asters at the poles of the dividing cell (Neujahr et al., 1998).

In multi-nucleate wild-type cells of *Dictyostelium discoideum* produced by electric-pulse-induced cell fusion, unilateral furrows are formed that by merging divide the cell body into multiple portions (Bindl et al., 2020). These data indicate that the mechanism of mitotic cleavage enables a cell to divide not only by a contractile ring (Satterwhite and Pollard, 1992) but also by the unilateral ingression of a furrow.

Here, we study unilateral furrow formation in a septase-knockout mutant of *D. discoideum* (Müller-Taubenberger et al., 2009). Septase (sepA) is a serine-threonine kinase homologous to Cdc7, a regulator of the septation-initiation network (SIN) in *Schizosaccharomyces pombe* (Feoktistova et al., 2012). In *D. discoideum*, septase is involved in controlling cell-to-substrate adhesion. In septase-null cells, cell-to-substrate adhesion is enhanced (Lampert et al., 2017), and the lifetime of pseudopods is prolonged (Singh et al., 2020). A substrate of septase is the Scar/WAVE complex, an activator of the Arp2/3 complex responsible for the generation of branched actin filament networks. Cell-to-substrate adhesion stimulates the phosphorylation of Scar/WAVE (Singh et al., 2020).

Cells of the septase-null mutant proved to be appropriate tools to study mitotic cleavage and, in particular, the ingression of unilateral furrows into multi-nucleate *Dictyostelium* cells. These mutant cells do not round up at the beginning of cytokinesis, and fewer furrows are formed in multi-nucleate cells than in a wild-type background, such that each furrow has more space to ingress into the large cell body (Müller-Taubenberger et al., 2009).

We show that in multi-nucleate cells, the initiation site and path of the unilateral furrows is programmed by a pattern of cortexillin (herein visualized by fluorescent cortexillin I) and myosin II laid out in the cortex by signals elicited from the microtubule system. Both proteins are depleted at the positions of microtubule asters, allowing the furrow to progress only at spaces that are free of the asters and consequently rich in cortexillin and myosin II.

## RESULTS

Since cell division is delayed in the septase-null mutant, patterns can develop on the substrate-attached surfaces of multi-nucleate cells before any furrow ingresses. We have confined cells between two parallel planar substrate surfaces and recorded the invasion of unilateral cleavage furrows in relation to the patterns formed by actin and by two proteins, cortexillin and myosin II, which in normal mitosis of mononucleate cells accumulate in the cleavage furrow.

Here, we present confocal recordings that exemplify characteristic features of protein patterns and cleavage furrows in multi-nucleate cells: first, the accumulation of actin in the area of microtubule asters and within protrusions of the cell where furrowing is prevented; second, the extension of a single furrow into an entire multi-nucleate cell; third, the depletion of cortexillin and myosin II at microtubule asters; and finally, an expanding ring-shaped furrow. In the following results, we show instructive examples of each type of labelled cell. These represent a total number of 89 recordings, as specified in Table S1.

### Dynamics of actin polymerization during unilateral furrow formation

**Fig. 1.**
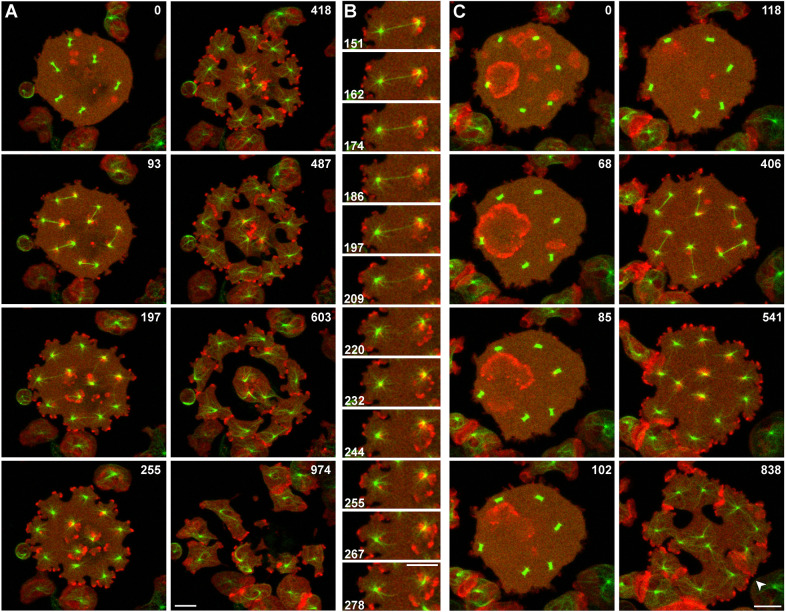
**Unilateral cleavage furrows in septase-null cells.** The cells expressed mRFP–LimEΔ as a label for filamentous actin (red) and GFP-α-tubulin (green). (A) A cell containing six nuclei, the synchronous division of which is visualized by the tubulin label. The actin label was strong in protrusions formed in regions where microtubule asters contacted the cell border and also in the vicinity of the asters on the substrate-attached cell surface. Cleavage furrows were initiated and progressed in the spaces between microtubule asters, independently of the presence or absence of a spindle in any of these spaces. This cell is also shown in Movie 1. (B) Section of the cell shown in A, highlighting the left-most mitotic complex, in which one microtubule aster is connected with the cell border, the other with the substrate-attached cell surfaces. (C) Disappearance of actin waves at the onset of mitosis. Propagation and extinction of an actin wave in this cell is seen in the frames between 0 s and 102 s. Entry of an actin-rich protrusion into a cleavage furrow is indicated in the 838 s frame by an arrowhead. This cell is also shown in Movie 2. Time is indicated in seconds after the first frame. Scale bars: 10 µm.

To visualize filamentous actin in relation to mitotic complexes, mRFP–LimEΔ (red) and GFP–α-tubulin (green) were expressed in septase-null cells (Fig. 1). The actin label was most prominent in the dynamic protrusions on top of the microtubule asters at the cell border, and also in mobile patches that emanated from the regions around centrosomes that were confined between the substrate surfaces (Fig. 1A,B; Movie 1).

The sequence shown in Fig. 1C and Movie 2 begins at a metaphase stage earlier than the one shown in Fig. 1A. Therefore, the disappearance of a propagating actin wave, typical of interphase cells, is captured (0–102 s frames of Fig. 1C). Otherwise, this sequence shows similar dynamics of actin in the course of mitosis as Fig. 1A, and in addition an often-observed feature, the entry of an actin-rich protrusion into a cleavage furrow (arrowhead in the 838 s frame of Fig. 1C).

In summary, actin forms short-lived patches in the centrosomal regions and is most abundant in cellular protrusions, reflecting its enrichment at the two poles of a normal dividing cell (Neujahr et al., 1997).

### Cortexillin pattern and the path of unilateral furrows

To explore two spatial relationships – the pattern of cortexillin depletion at the position of mitotic complexes and the invasion of cleavage furrows at sites of cortexillin accumulation – cells of the septase-null mutant were transfected to express GFP–cortexillin I (green) and RFP–α-tubulin (red) to visualize the mitotic complexes.

**Fig. 2.**
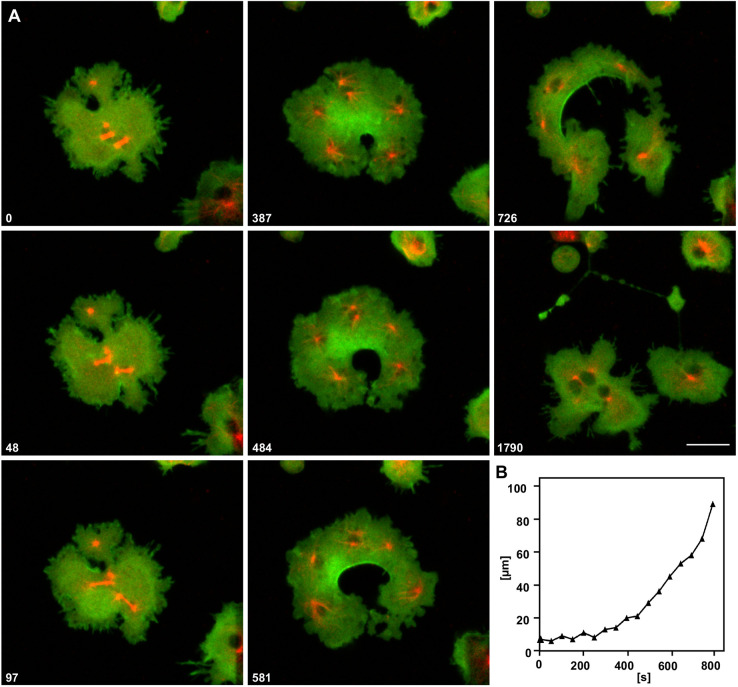
**Expansion of a unilateral cleavage furrow.** (A) A furrow ingressing at the bottom of the image and invading a cell entirely up to the top. The cell shown expressed RFP–α-tubulin to label the four mitotic complexes (red), and GFP–cortexillin I (green). Time is indicated in seconds after the first frame. Scale bar: 10 µm. (B) Plot showing the increase of contour length of the furrow during the course of its invasion. Steepening of the curve indicates expansion of the furrow into the cortexillin-rich area. Data are representative of five cells with just one furrow.

The undisturbed ingression of a unilateral cleavage furrow is shown in Fig. 2. The cell initially formed four furrows (48 s frame). Because three of them regressed, the only persisting furrow did not meet a counterpart. It rather expanded into the entire cortexillin-enriched area, assuming a mushroom-like shape (frames 387 s to 726 s), and progressed up to the opposite cell border where it turned a band of the cell into a delicate network (1790 s frame).

**Fig. 3.**
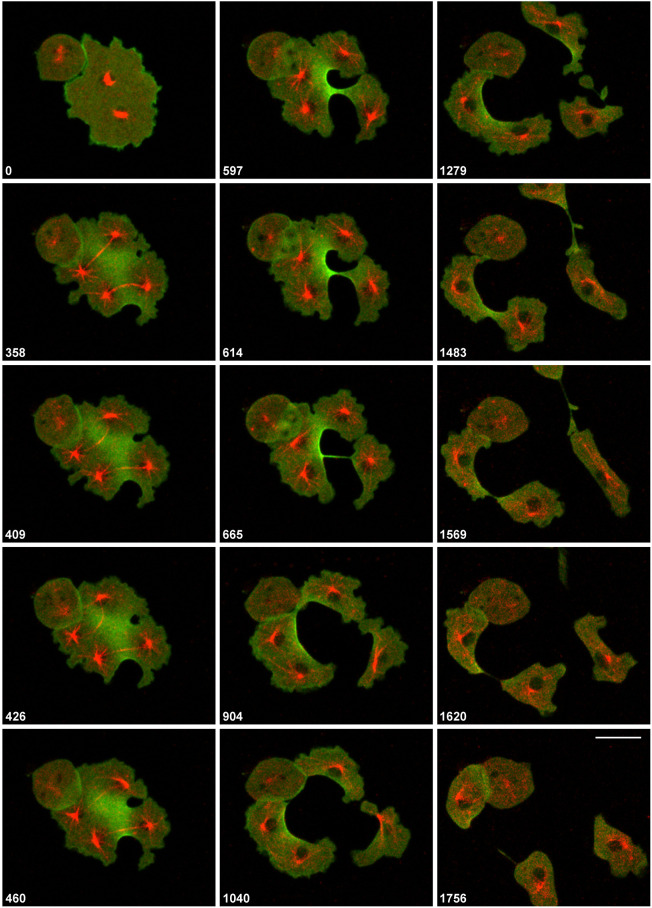
**A cell with two mitotic complexes as a link between divisions of mono- and multi-nucleate cells.** The cell shown expressed RFP–α-tubulin (red) and GFP–cortexillin I (green). Time is indicated in seconds after the first frame. This cell is also shown in Movie 3. Scale bar: 10 µm.

**Fig. 4.**
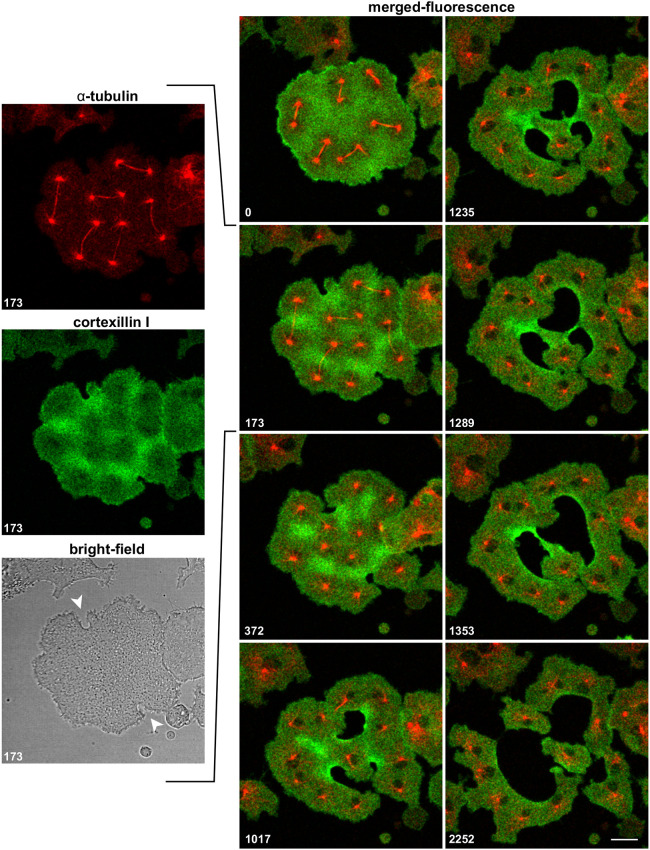
**A cell with six mitotic complexes.** The cell shown expressed RFP–α-tubulin (red) and GFP–cortexillin I (green). The time series shows merged fluorescence images of cortexillin depletion and cleavage furrow invasion. For the 173 s stage, the three recorded channels are shown separately on the left: the two fluorescence channels and the corresponding bright-field image. The two arrowheads in the latter point to incipient cleavage furrows. Time is indicated in seconds after the first frame. This cell is also shown in Movie 4. Scale bar: 10 µm.

A link between formation of a typical cleavage furrow in a mononucleate cell and the unilateral furrows formed in multi-nucleate cells is provided by the binucleate cell shown in Fig. 3 and Movie 3. Two furrows invaded this cell, the furrow at the bottom constricted together with the furrow on the right, similar to the furrow in a mononucleate cell. The furrow on the right separated two mitotic complexes from each other and participated in two cleavage events. A furrow that derived from fusion of the two primary furrows subsequently separated the remaining binucleate fragment into two mononucleate ones. The pattern of cortexillin enrichment is typical of multi-nucleate cells: it spares the zones controlled by microtubule asters surrounding the centrosomes, whereas the presence of a spindle does not prevent cortexillin enrichment. To show the expansion of furrows into the cortexillin-enriched areas more clearly, larger multinucleate cells were employed. Cortexillin accumulated at spaces not controlled by the microtubule asters that surrounded the centrosomes, such that circular areas around the centrosomes were distinguished by their low cortexillin content from the cortexillin-rich areas between these zones. When the microtubule asters moved, the areas of cortexillin depletion changed their position accordingly. This spatial relationship between the position of microtubule asters and cortexillin depletion is exemplified in Fig. 4 and Movie 4. The six mitotic complexes of the cell are labelled by RFP–α-tubulin (red; see 0 s frame). GFP–cortexillin (green) shows the depletion pattern around the mitotic complexes. During late anaphase, depletion was restricted to the regions of the microtubule asters surrounding the centrosomes, whereas the midzones on top of the spindles remained rich in cortexillin (173 s and 372 s frames).

Two unilateral furrows were initiated at cortexillin-rich sites of the cell border, one at the top, the other at the bottom of the image. The progression and expansion of both furrows followed the pattern of cortexillin enrichment. This is particularly clear for the lower furrow, which branched in order to extend into two arms of a cortexillin-rich field and to circumvent a microtubule aster. The furrow on top broadened where it entered a wide field of cortexillin enrichment. Eventually, both branches of the lower furrow fused with the upper furrow (frames 1289 s to 2252 s), generating joint furrows that expanded along propagating zones of cortexillin enrichment, pushing the microtubule asters ahead or separating asters from each other.

An often-observed midwifery activity is seen in the 1544 s to 1916 s frames of Movie 4, where an interphase cell pushes against the cleaving cell, resulting in final separation of the latter into four pieces.

The relationship of furrow invasion to areas of cortexillin accumulation is further illustrated by the cell shown in Movie 5. One furrow, probably stimulated by contact with another cell, invaded unusually early at the site of a spindle and branched into three lobes (370 s and 415 s frames). One of these lobes regressed (frames 582 s to 820 s), while the other two expanded further according to the pattern of cortexillin enrichment (frames 979 s to 1094 s). Subsequently, these two lobes joined each other, separating a cell with two centrosomes (fames 1341 s to 1402 s). Finally, while the joint furrow progressed in a wide arc, a portion of the cell with three centrosomes was cleaved off.

### Microtubule-independent and microtubule-dependent myosin II patterns

In normal mitosis, myosin II accumulates in the cleavage furrow as cortexillin does. To visualize myosin II patterns in large multi-nucleate cells, septase-null cells expressing GFP–myosin II heavy chain (green) and RFP–α-tubulin (red) were used. In cells gently compressed by an agarose overlay, there are two planar surfaces on which patterns can be recorded by confocal microscopy. On both surfaces, we observed, from anaphase onwards, a depletion of myosin II in the round areas on top of the microtubule asters, similar to the cortexillin-depleted areas. This local depletion was also observed on top of single centrosomes, which are neither associated with a nucleus nor do they form a spindle.

**Fig. 5.**
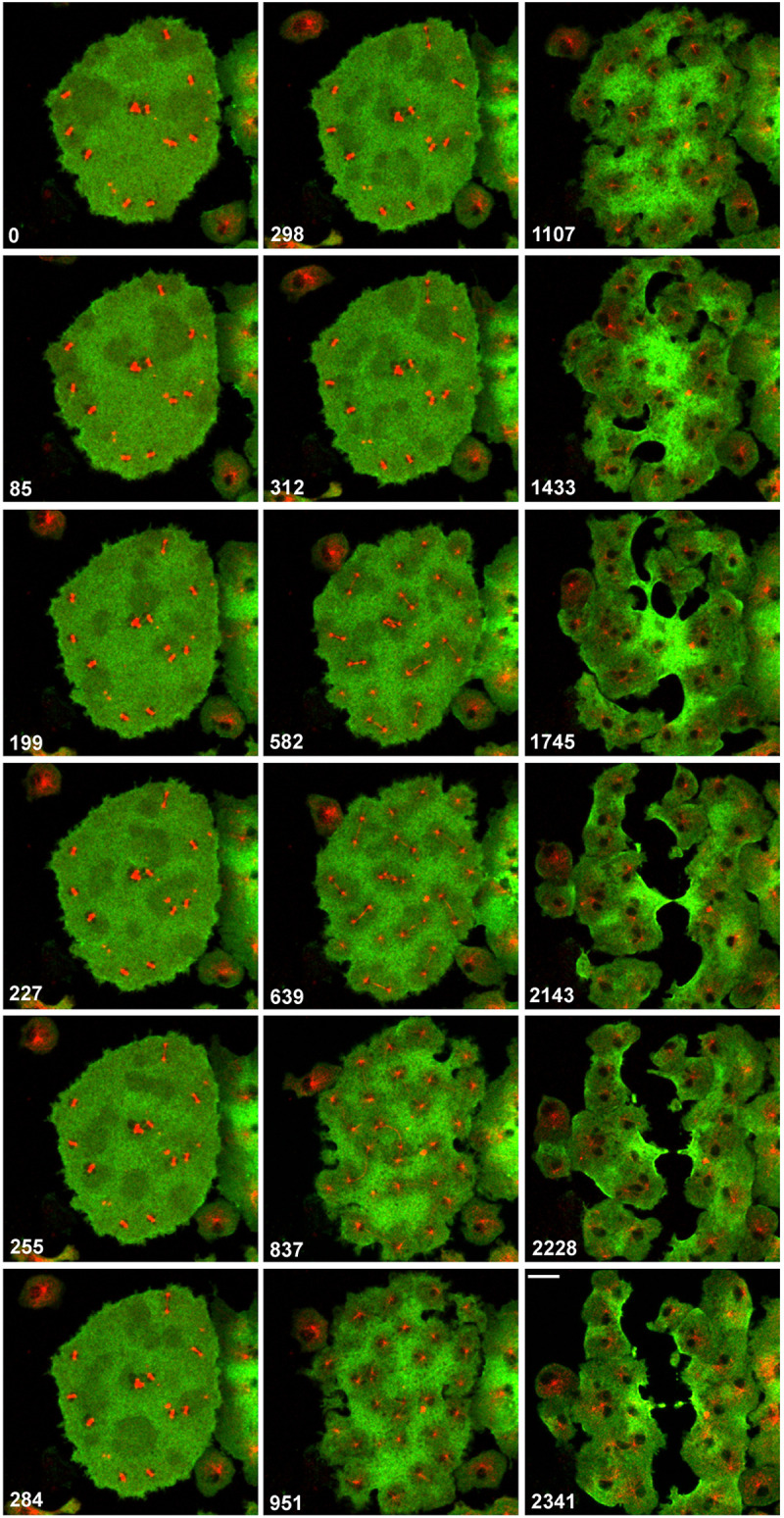
**Patterns of myosin II depletion during mitosis of a multi-nucleate cell.** RFP–α-tubulin is shown in red, and GFP–myosin II heavy chain is shown in green. Early stages show fluctuating areas of myosin II depletion that are independent of the positions of microtubule asters (frames 0 s to 312 s). During anaphase, myosin II became depleted in association with microtubule asters at the spindle poles (frames 639 s to 951 s). Multiple furrows ingressed into the myosin II-rich areas of the large cell and created a broad cleft by fusion (frames 2143 s to 2341 s). Time is indicated in seconds after the first frame. This cell is also shown in Movie 6. Scale bar: 10 µm.

Prior to the microtubule-linked pattern, an aster-independent myosin II pattern could be observed on the glass-attached cell surface: areas of myosin II depletion that increased up to a diameter of 15 µm. These negative fluctuations, which were independent of the positions of mitotic complexes, are shown in the metaphase stage of the large multi-nucleate cell in Fig. 5 (frames 0 s to 312 s) and Movie 6. These myosin II patterns correspond to actin waves that propagate on the substrate-attached cell surface during interphase (Gerhardt et al., 2014) and disappear early in mitosis (Fig. 1C; Movie 2). This view is supported by similar velocities of propagation. For the fronts of expanding myosin II depletions, we obtained a velocity of 4.5±0.8 µm/min (mean±s.d.; *n*=4); for the actin waves in early mitosis, we measured a velocity of 5.5±1.6 µm/min (mean±s.d.; *n*=5). This value is in accordance with the value of 6 µm/min published for actin waves in interphase cells (Bretschneider et al., 2009). During anaphase, these fluctuations were replaced by the circular low-myosin II zones embracing the microtubule asters (frames 639 s to 951 s in Fig. 5).

Shortly after the onset of the microtubule-dependent myosin II patterning, cleavage furrows began to ingress laterally at myosin II-rich sites of the membrane (Fig. 5, frames 837 s to 1107 s). The furrows followed a curled path through the myosin II-enriched areas of the membrane, thereby broadening at their front (frames 1433 s to 1745 s). Finally, the large cell was cut into portions by fusion of the furrows (frames 2143 s to 2341 s).

To summarize these data: both proteins, cortexillin and myosin II, which in mononucleate cells are recruited to the cleavage furrow, are linked in multi-nucleate cells to the planar pattern imprinted in anaphase by the microtubule asters on the substrate-attached cell surface.

### Increase of contour length of unilateral furrows invading into multi-nucleate cells

**Fig. 6.**
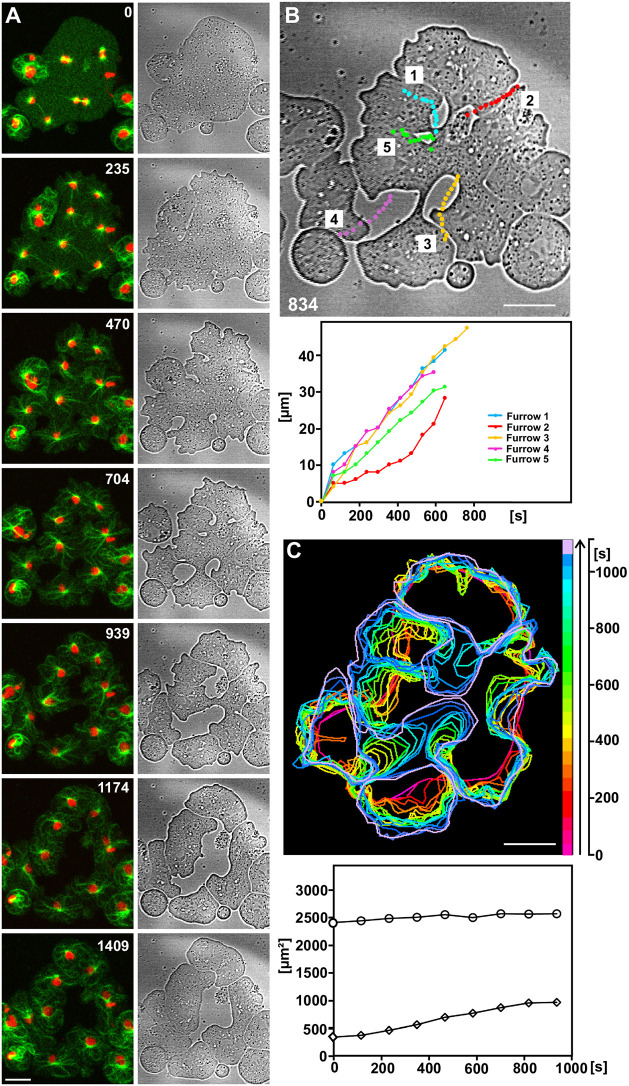
**Speed of invasion of five unilateral furrows and changes in surface area of a multi-nucleate cell.** (A) Left: a cell containing four mitotic complexes labelled with GFP–α-tubulin (green) and mRFP–histone 2B (red). Right: bright-field images showing division by the five unilateral furrows. Time is indicated in seconds after the first frame. (B) Top: paths of the tips of the five invading cleavage furrows superimposed on the image of the cell shown in A at the 834 s stage. Bottom: contour length of the five furrows as a function of time. (C) Top: contours of the same cell at consecutive times, colour-coded as indicated in the scale bar on the right. Bottom: surface area of the cell as a function of time. Circles: sum of the two planar substrate-attached areas, measured at the glass-attached surface and multiplied by a factor of 2. Diamonds: the free surface between the two substrate planes, approximated as a straight vertical contour. The height of the confined cell was estimated by confocal imaging to be 2.4 µm. Scale bars: 10 µm. Quantitative data for two more cells are shown in Fig. S1.

To quantify the changes in membrane area during the formation of unilateral furrows, we measured the contour lengths of the five furrows in a cell containing four mitotic complexes (Fig. 6A). A stage shortly before fusion of the furrows is displayed in the top panel of Fig. 6B, with the paths of the tips of the furrows indicated in colour. The contour lengths of the furrows during invasion are plotted in the lower panel. The corresponding shape changes of the cell during furrow invasion are shown in the upper panel of Fig. 6C. In the lower panel of Fig. 6C, the changes in cell surface area are plotted for the two substrate-attached surfaces and separately for the free surface in between. The latter increases during furrow invasion, as is obvious from the contours displayed in the upper panel.

Furrow 5 finally regressed, resulting in re-fusion of the portion of the cell separated by this furrow (frames 939 s to 1174 s). We repeatedly observed re-fusion of already separated parts of a cell, but never observed an attached interphase cell fusing with the dividing one, even when it came in close contact with the mitotic cell. These observations suggest that the high capacity to fuse is restricted to the mitotic stage.

### Expanding circular furrows

**Fig. 7.**
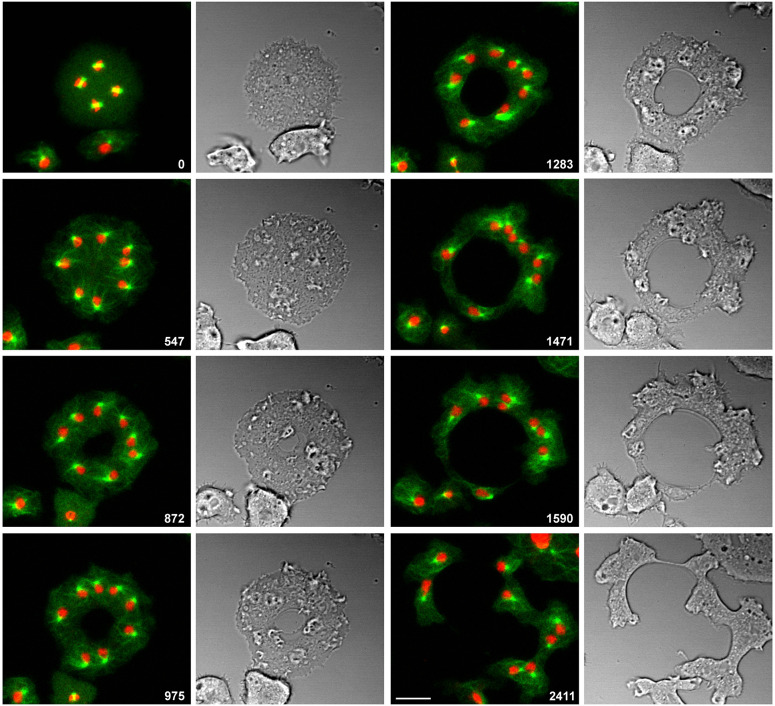
**An expanding ring-shaped cleavage furrow.** The cell shown contained four mitotic complexes, visualized using GFP–α-tubulin (green) to label the microtubule system and mRFP–histone 2B (red) to label the chromosomes. Fluorescence and bright-field images show furrow formation and division of the cell. Time is indicated in seconds after the first frame. Scale bar: 10 µm.

An obvious peculiarity of the furrows in the multi-nucleate cells is their expansion in the course of progression. This feature is most evident in the rare cases of a central furrow that expands as a circle. Such a furrow is shown in Fig. 7. In this case, the four mitotic complexes became located close to the cell border (frames 0 s to 547 s), apparently inhibiting the initiation of a furrow there, such that the centre of the cell remained the only space where a furrow could be initiated since it was free of microtubule asters (872 s frame). The central furrow expanded as a ring slightly asymmetrically (frames 975 s to 1471 s), until it left only thin cytoplasmic bridges between portions of the cell, which finally disrupted (frames 1590 s to 2411 s).

## DISCUSSION

The subject of the present study is the pattern formation in the cell cortex that underlies partitioning of a mitotic cell into cleavage furrow and polar regions. We explore the positioning of cleavage furrows in confined multi-nucleate cells of *Dictyostelium* and show that the ingression of these unilateral furrows is determined by a pattern in the cell cortex that is laid out by the microtubule asters. These radial arrays of microtubules are connected with their minus end to the centrosomes located at the two poles of each spindle, and extend with their plus end toward the cell cortex. Two proteins, myosin II and cortexillin, an anti-parallel actin-filament crosslinker (Faix et al., 1996), are depleted in the areas of the cell cortex that are controlled by the aster microtubules. In normal mononucleate cells, these proteins are localized to the cleavage furrow and are involved in its constriction (Fukui and Inoué, 1991; Weber et al., 1999; Yumura et al., 1984). These data are consistent with the finding that in epithelial mammalian cells, aster microtubules suppress Rho activity, and selective depolymerization of the asters results in lateral widening of the contractile ring (Murthy and Wadsworth, 2008).

The pattern of myosin II accumulation in the multi-nucleate cells of *Dictyostelium* changes in the course of anaphase. In the first phase, myosin II forms areas of depletion that are independent of the location of centrosomes or spindles. These areas expand with a velocity similar to the speed of actin waves propagating in interphase cells. Since the actin waves separate an internal myosin II-depleted area from a myosin II-rich area (Schroth-Diez et al., 2009) and cease at the beginning of mitosis (Fig. 1C; Movie 2), the myosin II fluctuations that are independent of mitotic complexes are proposed to reflect the propagation of these waves.

In interphase cells, actin waves have been shown to participate in the cytofission of *Dictostelium* cells (Flemming et al., 2020). In the light of these results, it is relevant that a role of these waves in cytokinesis is ruled out by their disappearance at the beginning of mitosis (Fig. 1C; Movie 2). In this respect *Dictyostelium* cells differ from *Xenopus* blastomeres, where waves of Rho activity and of actin polymerization co-exist with a cleavage furrow (Goryachev et al., 2016). Cytokinesis in *Dictostelium* is also distinct in terms of the role of the polar microtubule asters, rather than the central spindle, in determining the position of the cleavage furrow.

In the course of anaphase, the microtubule-independent myosin II fluctuations are replaced by a microtubule-dependent pattern: areas that are controlled by microtubule asters, which surround the centrosomes, become depleted of myosin II (Fig. 5). These aster-linked myosin II-depleted areas are long lived, and they change their position together with the underlying asters. The finding that, in addition to the filamentous myosin II, cortexillin is depleted in the areas of the microtubule asters, means that two proteins that in normal cytokinesis are recruited to the cleavage furrow display coinciding patterns in multi-nucleate cells.

The mechanism of cortexillin and myosin II localization remains to be clarified. The N-terminal motor domain of the myosin is not essential, supporting an actin-independent mechanism (Zang and Spudich, 1998). N-terminally truncated myosin II accumulates in the cytoplasmic space rather than in the cortex of the furrow region, which argues against a membrane-associated transport and supports the view of a microtubule-dependent mechanism (Vallee et al., 1990). In the case of *Dictyostelium*, this role could be attributed to the aster microtubules.

The mobile actin patches in the aster region on the substrate-attached cell surface resemble in their size, shape and short lifetime the protrusions at the cell border, in accordance with the notion that the aster areas correspond to the two polar regions of a dividing mononucleate cell (Fig. 1). These data complement the cortexillin and myosin II patterns in lending support to the view that the patterns we observe in multi-nucleate cells correspond to the distinction of polar regions and cleavage furrow in a mononucleate cell.

Notably, the cleavage furrows in multi-nucleate cells showed no particular enrichment of filamentous actin, corresponding to mononucleate cells where phalloidin staining shows the presence but no particular enrichment of filamentous actin in the furrow region (Neujahr et al., 1997; Weber et al., 1999). In this context, the actin-rich protrusions that are simultaneously formed with unilateral furrows may be relevant. In mammalian kidney epithelial cells, the local application of cytochalasin D to depolymerize actin selectively in the equatorial region does not inhibit the formation of a cleavage furrow. However, the application to a polar region does inhibit formation – an observation that led O'Connell et al. (2001) to suggest a global coordination of cortical activities in dividing cells.

**Fig. 8.**
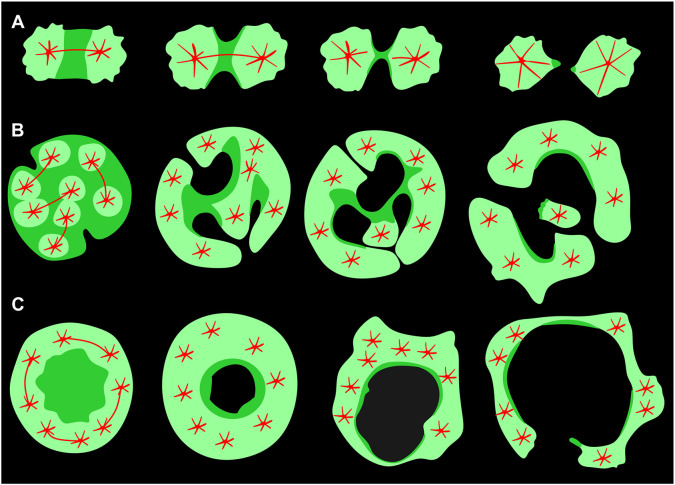
**Diagrams comparing the cortexillin patterns and cleavage furrow ingression in mononucleate and multi-nucleate cells.** Red, spindle and aster microtubules. Green, cortexillin. Stages of furrow progression are presented from left to right. (A) A dividing mononucleate cell showing cortexillin accumulation as a band in the equatorial region followed by constriction of the furrow. (B) A mitotic multi-nucleate cell forming unilateral furrows. In anaphase (left), cortexillin becomes depleted around the microtubule asters. Cleavage furrows ingress from the cell border at cortexillin-rich areas. The furrows branch and expand according to the shape of these areas, leaving out the aster regions. Eventually, the cell is divided into portions of irregular size at sites where two cleavage furrows meet and fuse. (C) A multi-nucleate cell forming an expanding circular cleavage furrow. When mitotic complexes are arranged near to the periphery of the cell, they leave a central area free for the enrichment of cortexillin. In this case, a cleavage furrow can be formed without any contact with the cell perimeter. Upon expanding, this single furrow partitions the cell into irregular portions.

In the diagram shown in Fig. 8, we compare the formation of cleavage furrows in relation to cortexillin patterns in mono- and multi-nucleate *Dictyostelium* cells. In a mononucleate cell, cortexillin accumulates in an equatorial ring, followed by constriction of that ring and the separation of two mononucleate daughter cells (Fig. 8A). Unilateral cleavage furrows in a multi-nucleate cell ingress at sites that are free of microtubule asters and consequently rich in cortexillin and myosin II. The furrows can branch and widen, and where two furrows fuse, pieces of the cell with undefined numbers of nuclei are separated (Fig. 8B). The finding that the path and shape of the furrows in the multi-nucleate cells are confined to the spaces where cortexillin and myosin II are enriched, relates the unilateral furrows to the canonical cleavage furrow of a mononucleate cell. Nevertheless, the geometry of the unilateral furrows differs from that of a normal, constricting cleavage furrow in the following respects: (1) The unilateral furrow does not form a contractile ring on its own; only when it meets another furrow does a constriction form that separates two portions of a cell. (2) On its way through the cell, the furrow expands, and its length does not seem to be limited. If there are no competing furrows, a furrow may transgress the entire multi-nucleate cell, as seen for the furrow in Fig. 2, which reached a contour length of 80 µm. (3) In a multi-nucleate cell, the furrow can even have the shape of an expanding ring (Figs 7 and 8C).

Despite these differences, the enrichment of cortexillin and myosin II suggests that the irregular-shaped areas between the microtubule asters displayed in confined multi-nucleate cells are equivalents of the cleavage furrow in a normal mononucleate cell, in accordance with a previous notion that mechanisms other than constriction of a contractile ring can bring about a furrow (Savoian et al., 1999).

Taken together, the patterns formed by cleavage furrows in multi-nucleate cells are consistent with the view that areas of the cell cortex enriched in myosin II and cortexillin have a contractile activity independent of their shape: in cytokinesis of a mononucleate cell, the ring-shaped cleavage furrow constricts (Pollard, 2020); in a motile interphase cell, the tail region retracts (Jay et al., 1995); and in case of the planar areas of multi-nucleate cells, contraction provides the space for unilateral or circular furrows to expand.

## MATERIALS AND METHODS

### Cell strains, culture conditions and sample preparation for confocal microscopy

For the expression of fluorescent proteins in the septase-null mutant (Müller-Taubenberger et al., 2009) derived from the AX2-214 strain of *D. discoideum*, cells were transfected with vectors encoding GFP–myosin II (Robinson et al., 2002), GFP–cortexillin I (Weber et al., 1999) pDRH-Hyg^R^:RFP–α-tubulin (Effler et al., 2006), GFP–α-tubulin (Neujahr et al., 1998), mRFP1–histone 2B (Bindl et al., 2020) or mRFPM–LimEΔ (Fischer et al., 2004). The dual fluorescently labelled strains and combinations of selection markers used are displayed in Table S1. The image series shown represent a bulk of 89 experiments as outlined in Table S1. Expanding ring furrows were observed in six cases.

Cells were cultivated in nutrient medium, as described by Malchow et al. (1972), supplemented with 10 µg/ml Blasticidin S (Gibco, Life Technologies Corporation, Grand Island, NY, USA), 10 µg/ml Geneticin (Sigma-Aldrich, St Louis, MO, USA) and/or 33 µg/ml Hygromycin B (Calbiochem, Merck KGaA, Darmstadt, Germany) in plastic Petri dishes at 21±2°C.

Two methods were used to increase the rate of mitotic stages in the multi-nucleate septase null-cells: (1) Cells were incubated overnight in LoFlo medium (ForMedium Ltd., Norfolk, UK) and the following day transferred onto HCl-cleaned cover-glass-bottom dishes (FluoroDish, WPI INC., Sarasota, FL, USA) with LoFlo medium (Samereier et al., 2010). Imaging began after 1–3 h. (2) Cells were rinsed off the Petri dish with 17 mM K^+^/Na^+^-phosphate buffer, pH 6.0, and transferred to an HCl-cleaned cover-glass-bottom dish. A 3 h incubation time in the phosphate buffer was followed by an incubation in LoFlo medium for 4–6 h.

For Figs. 1 and 7, large cells were produced by electric-pulse-induced fusion in 17 mM phosphate buffer, pH 6.0, as described by Gerhardt et al. (2014). After the fusion, cells were incubated for up to 6 h in LoFlo medium before imaging. All cells were overlaid by a thin agarose sheet (Fukui et al., 1987) when mitoses commenced.

### Confocal image acquisition and data processing

Confocal images were acquired on a Zeiss LSM 780 microscope equipped with a Plan-Apochromat 63×/NA 1.46 oil immersion or a Plan-Apochromat 40×/NA 1.2 water objective (Zeiss AG, Oberkochen, Germany). The images were processed using the image-processing package Fiji (http://Fiji.sc/Fiji) developed by Schindelin et al. (2012) on the basis of ImageJ (http://imagej.nih.gov/ij). In the fluorescence images, we show average projections of series of confocal planes. The plane-to-plane distance was 0.1–0.2 µm. Since the RFP label bleaches at long periods of imaging, we corrected the signal in the red channel with the Bleach Correction plugin in Simple Ratio Mode. Bright fields show single-plane phase-contrast images. The contour lengths of the furrows in Fig. 2B and Fig. 6B were measured with the Segmented Line Tool. In Fig. 6C, the Polygon Selection Tool was used for the colour-coded contours of the whole cell at consecutive time points, and the substrate-attached areas were calculated using the Analyze and Measure Tool.

## Supplementary Material

Supplementary information

Reviewer comments
