# Supplementary material for: Patterning of the cell cortex and the localization of cleavage furrows in multi-nucleate cells
Source: J Cell Sci. 2022 Apr 14;135(7):jcs259648. doi: 10.1242/jcs.259648 (PMC9016623; doi:10.1242/jcs.259648)
Supplement: Supplementary information [file joces-135-259648-s1.pdf]

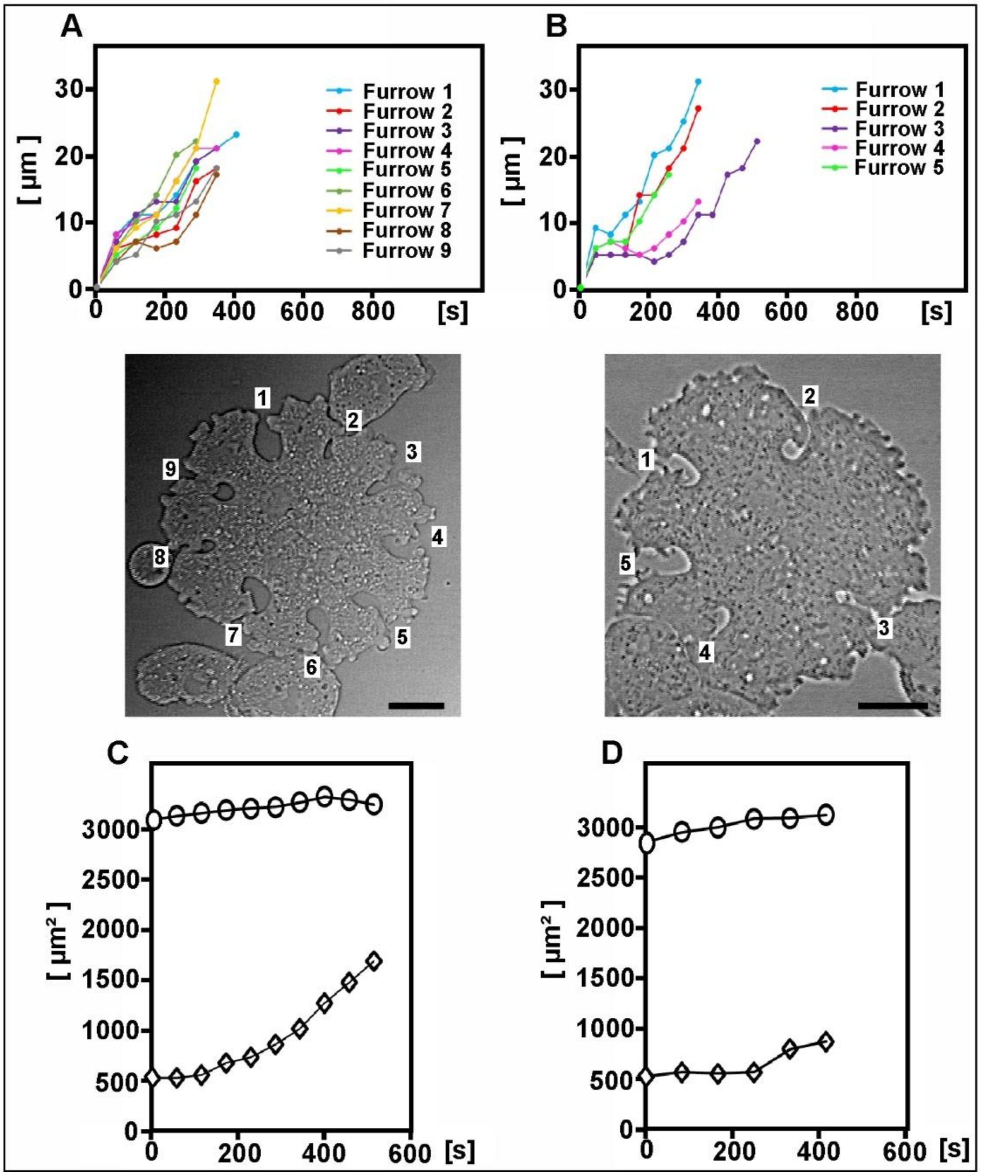

Fig. S1. Speed of invasion of unilateral furrows and changes in surface area of the multi-nucleate cells in Figure 1 A and C. A, B, increase in contour length of the unilateral furrows in the two cells. Furrow 8 in (A) and furrows 3, 4 in (B) proceeded to ingress after a period of stagnation. Furrows are indicated for (A) in the 418 s frame and for (B) in the 668 s frame corresponding to Figure 1 and Movies 1 and 2. Scale bars, 10 µm. **C, D,** surface area of the same two cells as a function of time. Circles: sum of the two planar substrate-attached areas. Diamonds: the free surface between the two substrate planes, approximated as a straight vertical contour. The height of the confined cell in (A, C) was estimated to be 3.4 µm, of the cell in (B, D) to be 2.8 µm.

Journal of Cell Science • Supplementary information

Table S1. Dual fluorescent labeled septase-null strains and number of multinucleate cells imaged in cytokinesis

GFP Label / RFP Label	Selection Markers	Number of cells
GFP-cortexillin I / pDRH-Hyg^R:RFP-α-tubulin	Geneticin / Hygromycin B	21
GFP-myosin II / pDRH-Hyg^R:RFP-α-tubulin	Geneticin / Hygromycin B	30
GFP-α-tubulin / mRFPM-LimEΔ	Geneticin / Hygromycin B	20
GFP-α-tubulin / mRFP1-histone 2B	Geneticin / Hygromycin B	18

All supplementary movies show average projections of confocal image series through confined Septase-null cells of *Dictyostelium discoideum*.

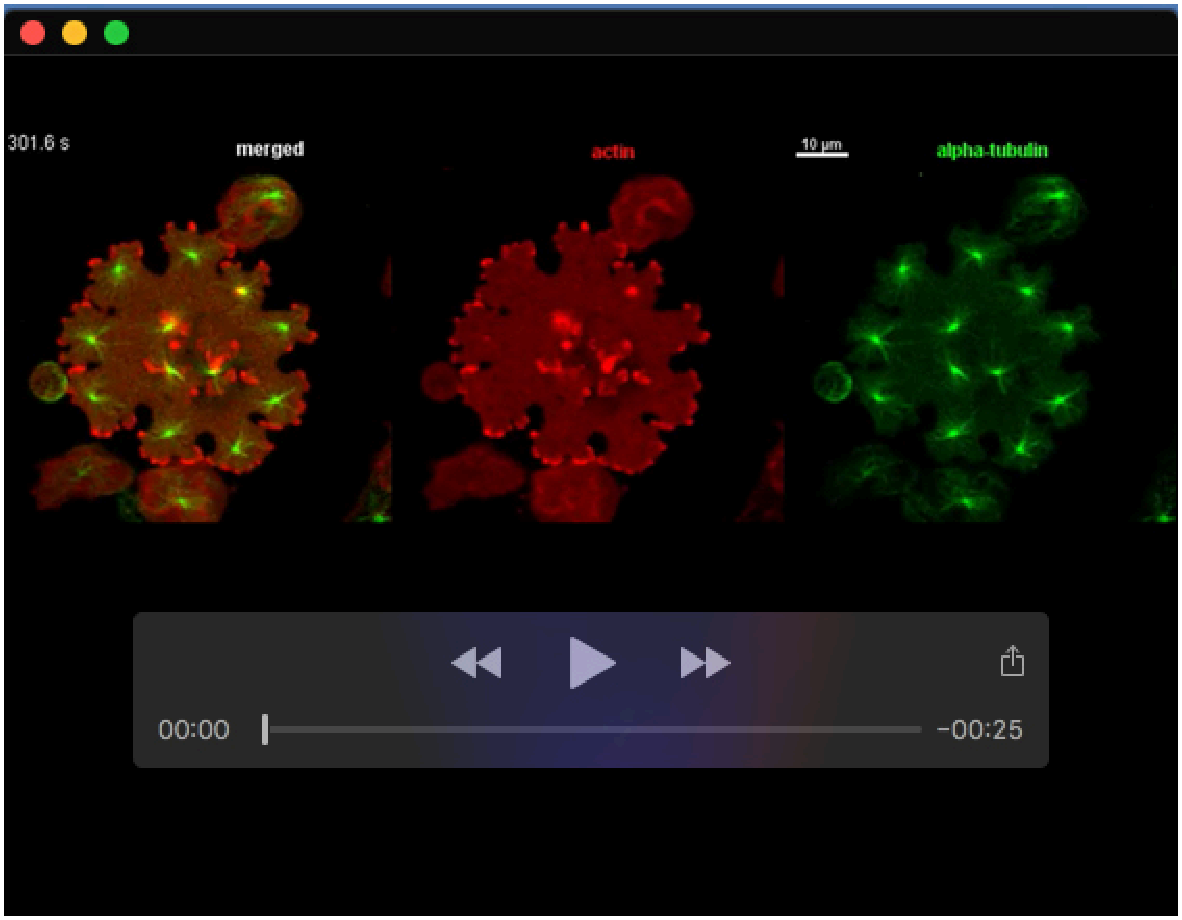

Movie 1. A septase-null cell that formed an unusually high number of nine unilateral furrows. The cell expressed GFP-α-tubulin to label centrosomes, spindles, and aster microtubules (green), and mRFP-LimEΔ as a label of filamentous actin (red). This movie is related to Figure 1A.

Journal of Cell Science • Supplementary information

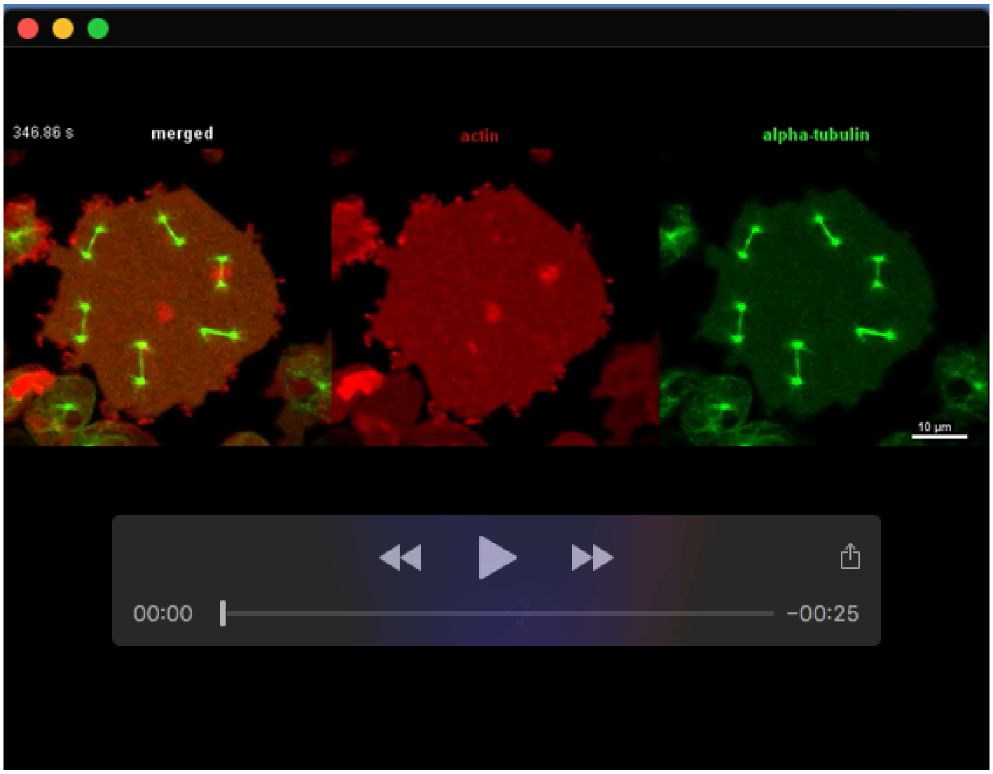

Movie 2. Disappearance of actin waves at the onset of mitosis. The cell expressed microtubule (green) and actin (red) markers as the cell in Movie 1. Propagation and extinction of an actin wave is seen in the 0 s to 102 s frames. Entry of an actin-rich protrusion into a cleavage furrow is indicated in the 838 s frame by an arrowhead. This movie is related to Figure 1C.

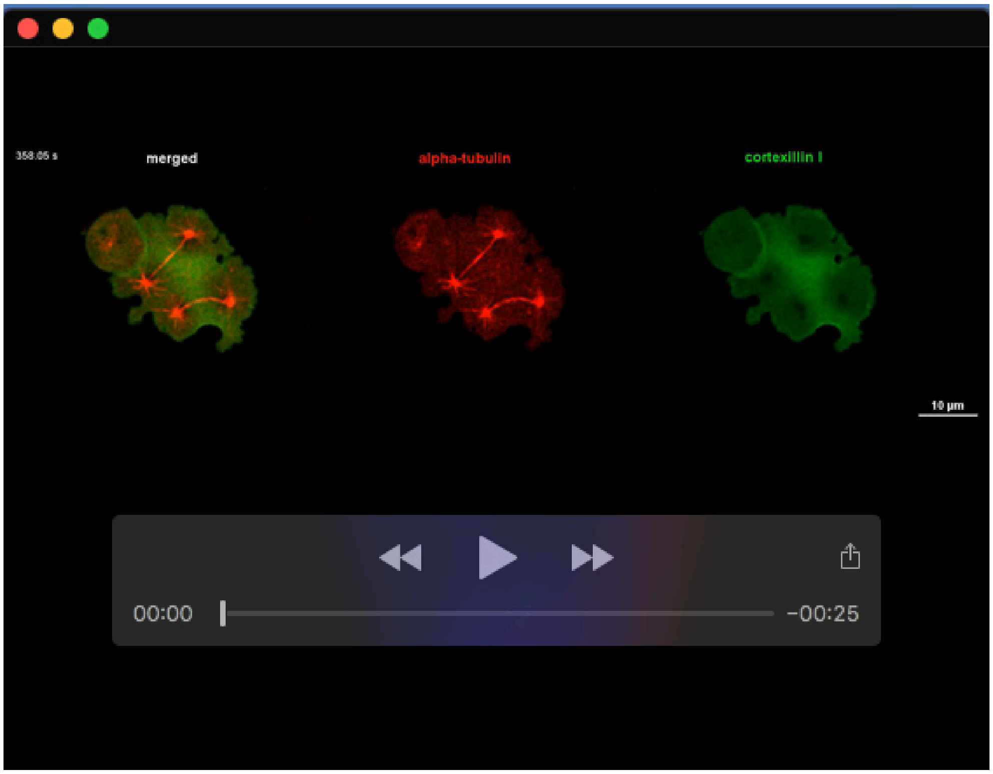

Movie 3. A binucleate septase-null cell showing two cleavage furrows that fused. The cell expressed RFP-α-tubulin (red) and GFP- cortexillin I (green). This movie is related to Figure 3.

Journal of Cell Science • Supplementary information

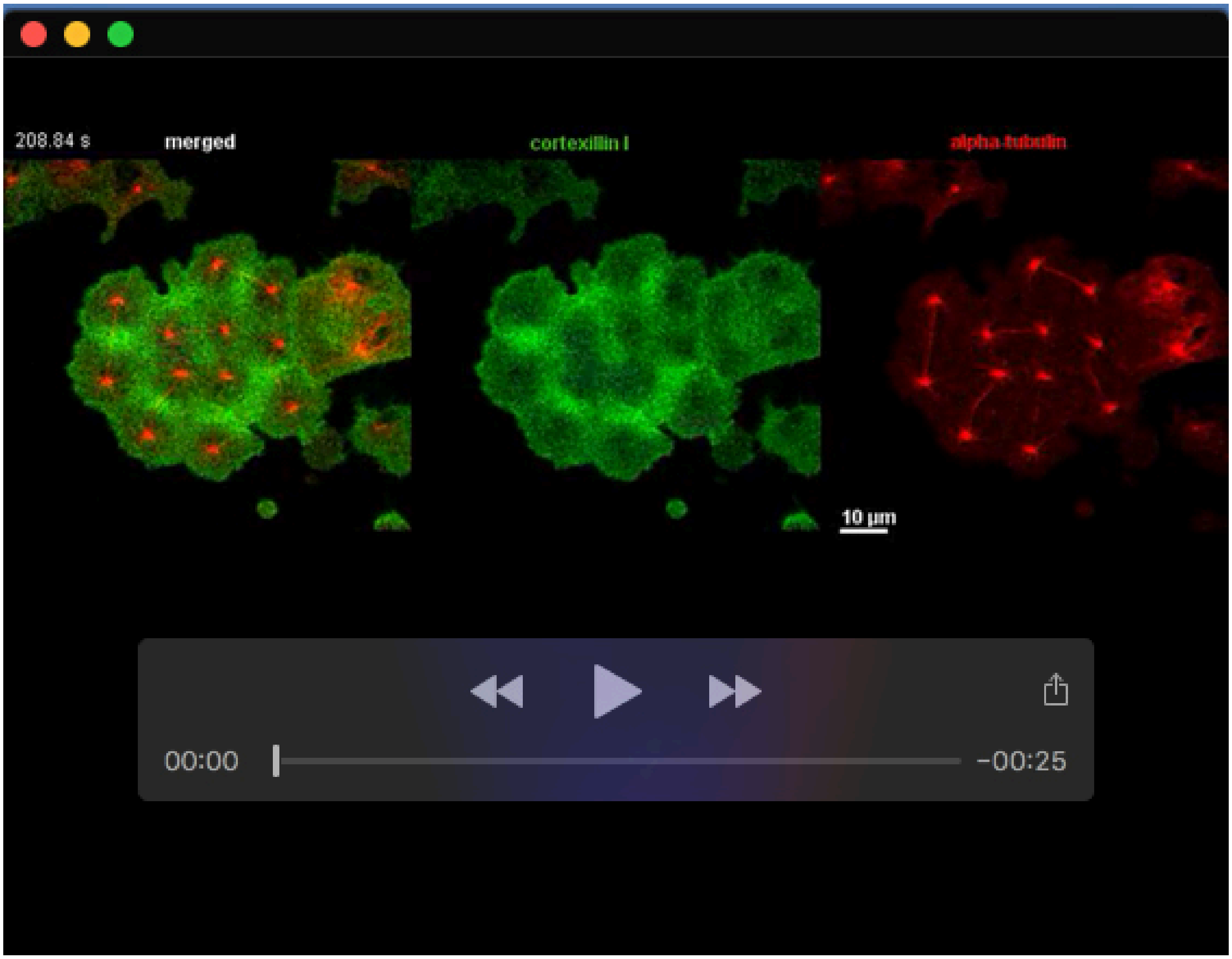

Movie 4. A septase-null cell that expressed RFP-α-tubulin (red) and GFP- cortexillin I (green) showing depletion of the cortexillin around the centrosomes. The cell formed two cleavage furrows that expanded into the cortexillin-rich area. Thereby the furrow at the bottom branched and eventually fused with the upper furrow. This movie is related to Figure 4.

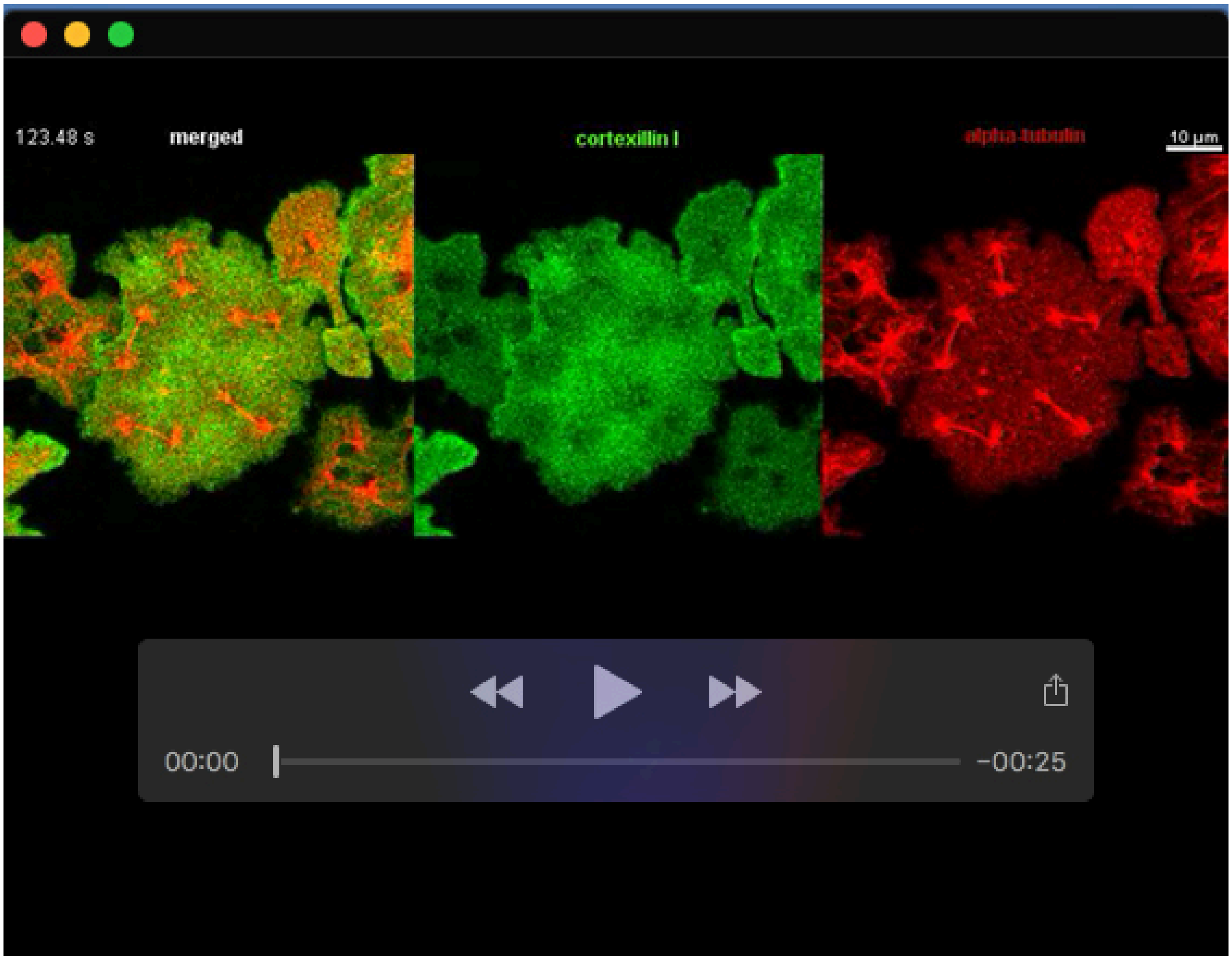

Movie 5. A septase-null cell invaded by a tri-lobed cleavage furrow. The cell expressed RFP-α-tubulin (red) and GFP- cortexillin I (green). The furrow entered the cell from the left side (370 s and 415 s frames), and two lobes continued to invade the cortexillin-rich area (820 s to 1341 s frames) until a binucleate portion was cleaved off (1402 s frame).

Journal of Cell Science • Supplementary information

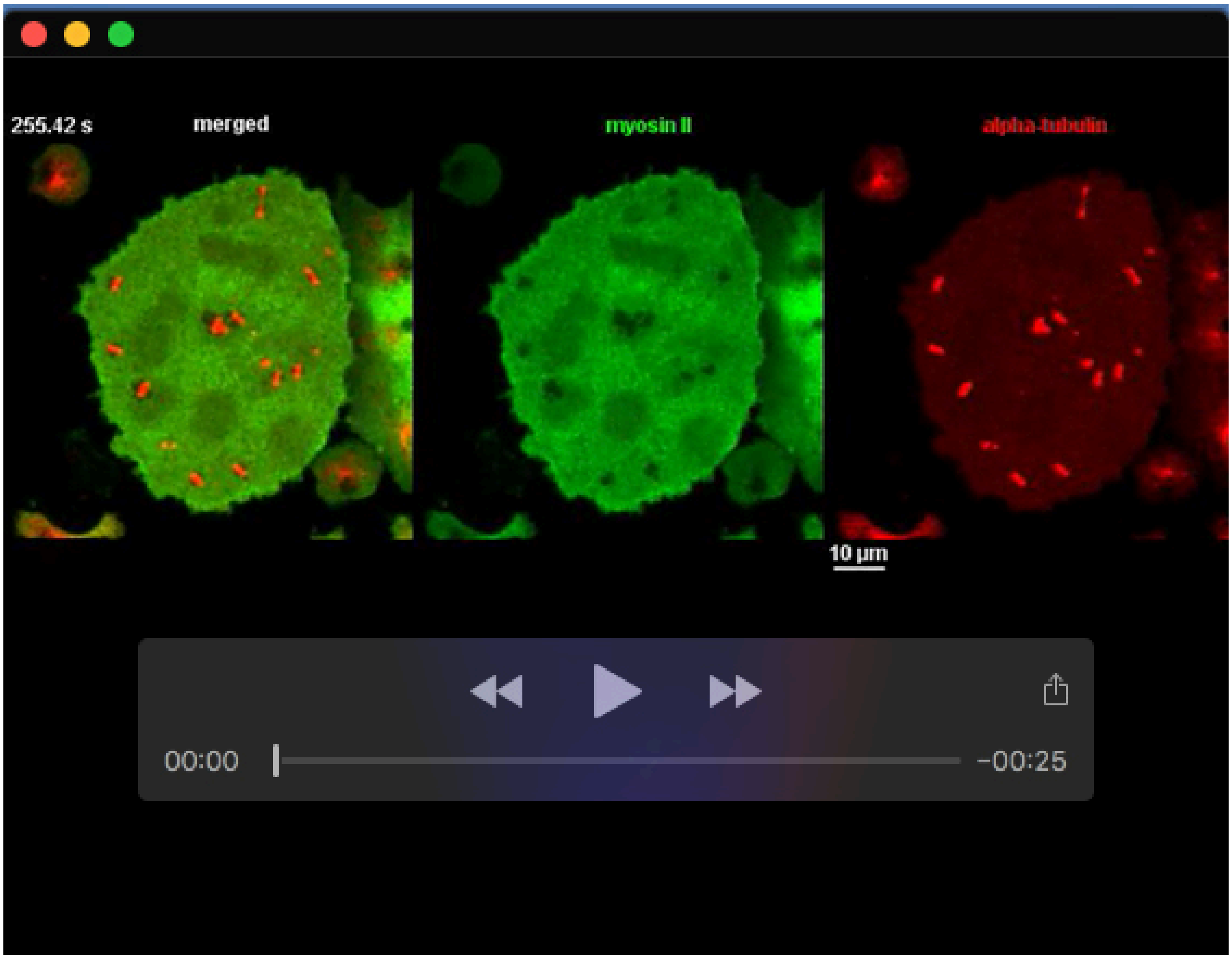

Movie 6. A septase-null cell that expressed RFP-α-tubulin (red) and GFP-myosin II heavy chains (green). The cell shows first wave-like depletions of myosin II that were independent of microtubule structures, and subsequently areas of depletion at the microtubule aster regions. These depletions are also seen around single centrosomes that are not connected by a spindle. This movie is related to Figure 5.

Journal of Cell Science • Supplementary information
