# Supplementary material for: Patterning of the cell cortex and the localization of cleavage furrows in multi-nucleate cells
Source: J Cell Sci. 2022 Apr 14;135(7):jcs259648. doi: 10.1242/jcs.259648 (PMC9016623; doi:10.1242/jcs.259648)
Supplement: Reviewer comments [file joces-135-259648_review_history.pdf]

Journal of Cell Science

Patterning of the cell cortex and the localization of cleavage furrows in multi-nucleate cells

Güenther Gerisch, Jana Prassler and Mary Ecke
DOI: 10.1242/jcs.259648

Editor: David Glover

Review timeline

Original submission:	1 December 2021
Editorial decision:	19 January 2022
First revision received:	11 February 2022
Accepted:	7 March 2022

Original submission

First decision letter

MS ID#: JOCES/2021/259648

MS TITLE: Patterning of the cell cortex and the localization of cleavage furrows in multi-nucleate cells

AUTHORS: Guenther Gerisch, Jana Prassler, and Mary Ecke
ARTICLE TYPE: Research Article

We have now reached a decision on the above manuscript.

To see the reviewers' reports and a copy of this decision letter, please go to: https://submit-jcs.biologists.org and click on the 'Manuscripts with Decisions' queue in the Author Area. (Corresponding author only has access to reviews.)

As you will see, the reviewers raise a number of substantial criticisms that prevent me from accepting the paper at this stage. They suggest, however, that a revised version might prove acceptable, if you can address their concerns. If you think that you can deal satisfactorily with the criticisms on revision, I would be pleased to see a revised manuscript. We would then return it to the reviewers.

We are aware that you may be experiencing disruption to the normal running of your lab that makes experimental revisions challenging. If it would be helpful, we encourage you to contact us to discuss your revision in greater detail. Please send us a point-by-point response indicating where you are able to address concerns raised (either experimentally or by changes to the text) and where you will not be able to do so within the normal timeframe of a revision. We will then provide further guidance. Please also note that we are happy to extend revision timeframes as necessary.

Please ensure that you clearly highlight all changes made in the revised manuscript. Please avoid using 'Tracked changes' in Word files as these are lost in PDF conversion.

I should be grateful if you would also provide a point-by-point response detailing how you have dealt with the points raised by the reviewers in the 'Response to Reviewers' box. Please attend to all of the reviewers' comments. If you do not agree with any of their criticisms or suggestions please explain clearly why this is so.

© 2022. Published by The Company of Biologists under the terms of the Creative Commons Attribution License (https://creativecommons.org/licenses/by/4.0/).

Reviewer 1

Advance summary and potential significance to field

Gerisch et al., investigated anaphase cortical patterning relevant for cleavage furrow formation in multi-nucleated Dictyostelium cells. Previous work demonstrated that Dictyostelium does not use spindle to position furrow, instead, cortexillin (a spectrin type protein) and myosin II act sequentially to define the site that eventually form furrow. Here using Septase-null mutant, which in addition to defects in cytokinesis also displayed increased cell adhesion, the authors found that a pattern of cortexillin and myosin II is generated on the anaphase cortex. The pattern of cortexillin formed due to a zone of depletion that correlated with the position of mitotic microtubule asters surrounding the centrosome. Even though the evidence is correlative, the visual effect of the mutual exclusion between aster and cortexillin is striking and very convincing. It is also a very interesting experimental system which potentially provides a physiological context to justify the dynamical as opposed to deterministic nature of the furrow positioning machinery. Therefore, I am in favor of its publication.

Comments for the author

I have a few questions before the manuscript is accepted. The author can address them either experimentally or through discussion.

1. Does LimE probe used for actin in this work label formin-nucleated actin filament? It is known that for instance GFP-actin does not label contractile rings. Alternatively, an immunofluorescence image of phalloidin can be shown to support a lack of actin in these furrows, unless it has been previously shown. After all, cortexillin is an actin bundling protein so the lack of actin is surprising.
2. In previous work of the same authors, a pattern of cortexillin gradient at the furrow was shown in WT cells or myosin II-null cells. It would be great to show them side-by-side in Figure 3, under the same imaging condition. Does cortexillin ventral pattern only appear in Septase-null mutant? Or were previous experiments imaged under a different imaging condition and missed such pattern (such as whether ventral plane or equatorial plane was imaged for confocal)?
3. I assume all the experiments were done by placing cells between two planal surfaces. The authors previously showed nicely that this led to oscillating waves alternating between dorsal and ventral surfaces for interphase cells (Helenius et al., 2018). If asters send our inhibitory signals, ventral and dorsal pattern should register, instead of oscillating in phase. Is that the case? Secondly, is confinement a necessary condition? If these large cells are adherent, would unilateral furrowing and cortexillin pattern take place without the confinement.
4. It would be great to show some frames before time 358 in Fig 4 (or a movie). The pattern of cortexillin is already fully formed by frame 358. What is the spatial relation between the site of their initiation and spindle or aster?
5. In Fig 4, one of the furrows is a traditional furrow (where the spindle center was) and the other is a Rappaport furrow. Are there any noticeable differences between them in this experimental system? In Figure 5 and Supplemental Movie 3, it seems like cortexillin signals dynamically changing from marking Rappaport furrow first to traditional furrow later. Is this strictly distance-dependent?
6. Because cortexillin patterns are dynamic, it seems like not all sites marked with cortexillin proceed to successful furrowing. For instance, only 1 of the 6 spindles in Movie 3 succeeded. Therefore, it appears cortexillin pattern is necessary but not sufficient. It remains curious what is the deciding factor for the success of cleaving.
7. Does the inhibition effect of aster require direct contact of microtubule with the cortex? The propagating pattern described here seems reminiscent of the wandering furrow described in other system, but usually under nocodazole treatment (Murthy 2008; Zhou 2008; Savoian 1999). If that is also true in this system, it would suggest that while there are correlation with asters, direct contact is not necessary.
8. One of the major conclusions is that “Unilateral furrows are distinguished from the contractile ring of a normal furrow by their expansion rather than constriction.” I wonder whether it is accurate to conclude so. Expansion of furrowing activity (or potential activity) does not necessarily imply a lack of local contraction. It appears to me that they describe two separate aspects of the process, one is on the biochemical patterning machinery while the other is on the mechanical process which is likely more downstream.

© 2022. Published by The Company of Biologists under the terms of the Creative Commons Attribution License (https://creativecommons.org/licenses/by/4.0/).

Reference:
1. Murthy, K. & Wadsworth, P. Dual role for microtubules in regulating cortical contractility during cytokinesis. J Cell Sci 121, 2350–2359 (2008).
2. Zhou, M. & Wang, Y.-L. Distinct pathways for the early recruitment of myosin II and actin to the cytokinetic furrow. Mol Biol Cell 19, 318–326 (2008).
3. Savoian, M. S., Khodjakov, A. & Rieder, C. L. Unilateral and wandering furrows during mitosis in vertebrates: implications for the mechanism of cytokinesis. Cell Biology International 23, 805–812 (1999).

Reviewer 2

Advance summary and potential significance to field

In this MS, the authors described dynamic localization of F-actin, cortexillin and myosin during the formation of cleavages furrows in multi-nucleate Dictyostelium cells (Septase-null cells). Using a time-lapse fluorescence microscope, they observed dynamics of actin polymerization (Figure 1 and 2), location of cortexillin (Figure 3 and 4), distribution of myosin II (Figure 5 and 6), changes in membrane area (Figure 7 and 8) during the furrow formation in multi-nucleate cells. The observations and measurements of dynamic changes in these proteins are very interesting and would contribute to our understanding mechanisms underlying the initiation and progression of the cleavage furrows to complete the cell division. The imaging data presented in the MS are high quality.
I suggest the following to improve the MS during the revision 1. The authors should present a graph model to summarize their observations. A model may help readers to understand the fundamental question in the field and conclusions of this study to the field.
2. The authors may present current views regarding the mechanisms that control the formation and completion of the cleavage furrows in other organisms and compare those views with their discoveries in Dictyostelium cells.
In conclusion, the MS presented novel observations, and it could be an interesting MS to not only a few in the field but also a broader audience with some changes.

Comments for the author

I suggest the following to improve the MS during the revision 1. The authors should present a graph model to summarize their observations. A model may help readers to understand the fundamental question in the field and conclusions of this study to the field.
2. The authors may present current views regarding the mechanisms that control the formation and completion of the cleavage furrows in other organisms and compare those views with their discoveries in Dictyostelium cells.
In conclusion, the MS presented novel observations, and it could be an interesting MS to not only a few in the field but also a broader audience with some changes.

First revision

Author response to reviewers' comments

Response to Reviewer 1

1. *Does LimE probe used for actin in this work label formin-nucleated actin filament? It is known that for instance GFP-actin does not label contractile rings. Alternatively, an immunofluorescence image of phalloidin can be shown to support a lack of actin in these furrows, unless it has been previously shown. After all, cortexillin is an actin bundling protein so the lack of actin is surprising.*

   The LimE probe labels the front of actin waves, where formin B is localized (Ecke et al., 2020, Mol. Biol. Cell. 31:373-385). Phalloidin has been used to label dividing *Dictyostelium* cells

© 2022. Published by The Company of Biologists under the terms of the Creative Commons Attribution License (https://creativecommons.org/licenses/by/4.0/).

and shown that there is no particular accumulation of actin (Neujahr et al., 1997; Weber et al., 1999). We have now made clear in the Discussion, page 10, that we do not claim the absence of F-actin from the cleavage furrow, but only say that there is no enrichment.

2. *In previous work of the same authors, a pattern of cortexillin gradient at the furrow was shown in WT cells or myosin II-null cells. It would be great to show them side-by-side in Figure 3, under the same imaging condition. Does cortexillin ventral pattern only appear in Septase- null mutant? Or were previous experiments imaged under a different imaging condition and missed such pattern (such as whether ventral plane or equatorial plane was imaged for confocal)?*

   Previously, equatorial planes were imaged, where the cortexillin pattern is not seen. The depletion of cortexillin from the aster regions is also seen in wild-type and myosin II-null cells.

3. *I assume all the experiments were done by placing cells between two planal surfaces. The authors previously showed nicely that this led to oscillating waves alternating between dorsal and ventral surfaces for interphase cells (Helenius et al., 2018). If asters send our inhibitory signals, ventral and dorsal pattern should register, instead of oscillating in phase. Is that the case? Secondly, is confinement a necessary condition? If these large cells are adherent, would unilateral furrowing and cortexillin pattern take place without the confinement.*

   The astral patterns did not oscillate. The previously reported surface switching applied to actin waves. As we report in the present manuscript, these waves disappear at the beginning of mitosis.

4. *It would be great to show some frames before time 358 in Fig 4 (or a movie). The pattern of cortexillin is already fully formed by frame 358. What is the spatial relation between the site of their initiation and spindle or aster?*

   As the reviewer suggested, we show now additional frames before 358 s of Figure 4 in a new Movie 3.

5. *In Fig 4, one of the furrows is a traditional furrow (where the spindle center was) and the other is a Rappaport furrow. Are there any noticeable differences between them in this experimental system? In Figure 5 and Supplemental Movie 3, it seems like cortexillin signals dynamically changing from marking Rappaport furrow first to traditional furrow later. Is this strictly distance-dependent?*

   Distances between two centrosomes belonging to one mitotic complex are limited by the length of the spindle. If two centrosomes are not connected by a spindle, their distance can be larger; consequently, the ingression of a furrow is favored at this space.

6. *Because cortexillin patterns are dynamic, it seems like not all sites marked with cortexillin proceed to successful furrowing. For instance, only 1 of the 6 spindles in Movie 3 succeeded. Therefore, it appears cortexillin pattern is necessary but not sufficient. It remains curious what is the deciding factor for the success of cleaving.*

   The scarce furrowing is one reason why we have chosen Septase-null cells for this study. It may be the quantity of cortexillin and myo-II accumulation rather than an additional factor that determines whether or not a furrow progresses.

7. *Does the inhibition effect of aster require direct contact of microtubule with the cortex? The propagating pattern described here seems reminiscent of the wandering furrow described in other system, but usually under nocodazole treatment (Murthy 2008; Zhou 2008; Savoian 1999). If that is also true in this system, it would suggest that while there are correlation with asters, direct contact is not necessary.*

   We have now added a reference to the paper by Zang and Spudich (1998) to the Discussion, which reports that a truncated myosin II that does not bind actin, is transported to the

© 2022. Published by The Company of Biologists under the terms of the Creative Commons Attribution License (https://creativecommons.org/licenses/by/4.0/).

cleavage furrow and accumulates there in the cytoplasm. In addition, we refer to a paper by Vallee, who emphasized transport along microtubules. In the light of these data, association with the cell cortex or the membrane is not required for the translocation.

8. *One of the major conclusions is that "Unilateral furrows are distinguished from the contractile ring of a normal furrow by their expansion rather than constriction." I wonder whether it is accurate to conclude so. Expansion of furrowing activity (or potential activity) does not necessarily imply a lack of local contraction. It appears to me that they describe two separate aspects of the process, one is on the biochemical patterning machinery while the other is on the mechanical process which is likely more downstream.*

   We agree that we deal with two aspects, patterning and the mechanical process of furrowing. Also in our view, the latter will involve local contraction. Nevertheless, it appears obvious that the extreme expansion of a furrow, as shown in Figures 2 and 7, differs from the typical constriction of a ring. We now extend on this point in the Discussion.

We thank the Reviewer for suggesting additional references; we refer now to the papers by Murthy and Wadsworth (2008), by Savoian et al. (1999), and have added some more references.

Response to Reviewer 2

1. *The authors should present a graph model to summarize their observations. A model may help readers to understand the fundamental question in the field and conclusions of this study to the field.*

   We have now added, as the new Figure 8, a graph comparing a typical constricting furrow in a mono-nucleate cell with an unilateral and an expanding ring-shaped furrow in multi-nucleate cells.

2. *The authors may present current views regarding the mechanisms that control the formation and completion of the cleavage furrows in other organisms and compare those views with their discoveries in Dictyostelium cells. In conclusion, the MS presented novel observations, and it could be an interesting MS to not only a few in the field but also a broader audience with some changes.*

   We refer now at several additional places in the Discussion (new passages labeled red) to the literature on other organisms, as far as they are directly related to our data.

Second decision letter

MS ID#: JOCES/2021/259648

MS TITLE: Patterning of the cell cortex and the localization of cleavage furrows in multi-nucleate cells

AUTHORS: Guenther Gerisch, Jana Prassler, and Mary Ecke
ARTICLE TYPE: Research Article

I am happy to tell you that your manuscript has been accepted for publication in Journal of Cell Science, pending standard ethics checks.

Reviewer 1

Advance summary and potential significance to field

The authors have addressed all my questions and I am happy to support it's publication.

© 2022. Published by The Company of Biologists under the terms of the Creative Commons Attribution License (https://creativecommons.org/licenses/by/4.0/).

Comments for the author

n/a

Reviewer 2

Advance summary and potential significance to field

The authors have addressed my concerns, and I support the revised MS to be published in JCS.

Comments for the author

I support the revised MS to be published in JCS.

© 2022. Published by The Company of Biologists under the terms of the Creative Commons Attribution License (https://creativecommons.org/licenses/by/4.0/).
